# Prostaglandin D_2_-supplemented “functional eicosanoid testing and typing” assay with peripheral blood leukocytes as a new tool in the diagnosis of systemic mast cell activation disease: an explorative diagnostic study

**DOI:** 10.1186/s12967-014-0213-2

**Published:** 2014-08-12

**Authors:** Dirk Schäfer, Peter Dreßen, Stefan Brettner, Norbert-Folke Rath, Gerhard J Molderings, Katrin Jensen, Christina Ziemann

**Affiliations:** Medical Clinic III, Friedrich-Alexander-University Erlangen-Nuremberg, Erlangen, Germany; Department of Internal Medicine, St. Franziskus Hospital, Eitorf, Germany; Department of Oncology, Haematology and Palliative Care, District Hospital of Waldbröl, Waldbröl, Germany; Oststadt-Heidehaus Hospital Laboratory, Klinikum Region Hannover (clinical centre of the Hannover region), Hannover, Germany; Institute of Human Genetics, University Hospital of Bonn, Bonn, Germany; Institute of Medical Biometry and Informatics, University Heidelberg, Heidelberg, Germany; Fraunhofer Institute for Toxicology and Experimental Medicine ITEM, Hannover, Germany

**Keywords:** Systemic mast cell activation disease, Eicosanoids, PGE_2_, PGD_2_, Peptido-leukotrienes, Functional eicosanoid testing and typing, Peripheral blood leukocytes, Substance P, Acetylsalicylic acid, Arachidonic acid

## Abstract

**Background:**

Systemic mast cell activation disease (MCAD) is characterized by an enhanced release of mast cell-derived mediators, including eicosanoids, which induce a broad spectrum of clinical symptoms. Accordingly, the diagnostic algorithm of MCAD presupposes the proof of increased mast cell mediator release, but only a few mediators are currently established as routine laboratory parameters. We thus initiated an explorative study to evaluate *in vitro* typing of individual eicosanoid pattern of peripheral blood leukocytes (PBLs) as a new diagnostic tool in MCAD.

**Methods:**

Using the “functional eicosanoid testing and typing” (FET) assay, we investigated the balance (i.e. the complex pattern of formation, release and mutual interaction) of prostaglandin E_2_ (PGE_2_) and peptido-leukotrienes (pLT) release from PBLs of 22 MCAD patients and 20 healthy individuals. FET algorithms thereby consider both basal and arachidonic acid (AA)-, acetylsalicylic acid (ASA)-, and substance P (SP)-triggered release of PGE_2_ and pLT. The FET assay was further supplemented by analyzing prostaglandin D_2_ (PGD_2_), as mast cell-specific eicosanoid.

**Results:**

We observed marked PGE_2_-pLT imbalances for PBLs of MCAD patients, as indicated by a markedly enhanced mean FET value of 1.75 ± 0.356 (range: 1.14–2.36), compared to 0.53 ± 0.119 (range: 0.36-0.75) for healthy individuals. In addition, mean PGD_2_ release from PBLs of MCAD patients was significantly, 6.6-fold higher than from PBLs of healthy individuals (946 ± 302.2 pg/ml versus 142 ± 47.8 pg/ml; *P* < 0.001). In contrast to healthy individuals, PGD_2_ release from PBLs of MCAD patients was markedly triggered by SP (mean: 1896 ± 389.7 pg/ml; *P* < 0.001), whereas AA and ASA caused individually varying effects on both PGD_2_ and pLT release.

**Conclusions:**

The new *in-vitro* FET assay, supplemented with analysis of PGD_2_, demonstrated that the individual patterns of eicosanoid release from PBLs can unambiguously distinguish MCAD patients from healthy individuals. Notably, in our analyses, the FET value and both basal and triggered PGD_2_ levels were not significantly affected by MCAD-specific medication. Thus, this approach may serve as an *in-vitro* diagnostic tool to estimate mast cell activity and to support individualized therapeutic decision processes for patients suffering from MCAD.

**Electronic supplementary material:**

The online version of this article (doi:10.1186/s12967-014-0213-2) contains supplementary material, which is available to authorized users.

## German abstract

**Hintergrund:**

Die systemische Mastzellaktivierungserkrankung (MCAD) ist durch eine vermehrte Freisetzung von Mastzellmediatoren gekennzeichnet, die für ein sehr breites Spektrum an klinischen Symptomen in MCAD Patienten verantwortlich sind. Das diagnostische Vorgehen bei MCAD erfordert den Nachweis einer erhöhten Mediatorfreisetzung, wobei aber bisher nur eine begrenzte Anzahl an Mediatoren als Routinelaborparameter etabliert ist. Es wurde daher eine Machbarkeitsuntersuchung initiiert, um die *in vitro* Bestimmung patientenspezifischer Eicosanoidmuster an peripheren Blutleukozyten als neue diagnostische Möglichkeit bei MCAD zu evaluieren.

**Methode:**

Analysiert wurde das Gleichgewicht der Freisetzung zwischen Prostaglandin E_2_ (PGE_2_) und Peptidleukotrienen (pLT) aus peripheren Blutleukozyten von 22 MCAD Patienten und 20 gesunden Personen als Kontrollen. Hierzu wurde die etablierte Methode der “funktionellen Eicosanoidtestung und -typisierung” (FET) eingesetzt, deren Auswertealgorithmus sowohl die basale als auch die Arachidonsäure (AA)-, Acetylsalicylsäure (ASA)- und Substanz P (SP)-stimulierte Freisetzung von PGE_2_ und pLT berücksichtigt. Zusätzlich wurde die Freisetzung von Prostaglandin D_2_ (PGD_2_) analysiert, da PGD_2_ als ein für Mastzellen relativ spezifisches Eicosanoid angesehen wird.

**Ergebnisse:**

Verglichen mit der Kontrollgruppe (mittlerer FET Wert: 0.53 ± 0.119; Spannweite: 0.36-0.75) wurden für MCAD Patienten deutliche PGE_2_-pLT Ungleichgewichte detektiert (mittlerer FET Wert: 1.75 ± 0.356; Spannweite: 1.14–2.36), wobei ein höherer FET Wert ein größeres Ungleichgewicht anzeigt. Zusätzlich war die mittlere PGD_2_ Freisetzung aus PBLs von MCAD Patienten signifikant 6.6-fach höher als in der Kontrollgruppe (946 ± 302.2 pg/ml versus 142 ± 47.8 pg/ml; *P* < 0.001). Im Gegensatz zur Kontrollgruppe war die PGD_2_ Freisetzung bei MCAD Patienten signifikant durch SP stimulierbar (Mittelwert: 1896 ± 389.7 pg/ml; *P* < 0.001), wohingegen AA und ASA sehr individuell variierende Effekte auf die PGD_2_ und pLT Freisetzung induzierten.

**Schlussfolgerungen:**

Der durch PGD_2_ ergänzte *in-vitro* FET Test war in der Lage, MCAD Patienten aufgrund der individuellen Eicosanoid-Freisetzungsmuster ihrer PBLs eindeutig von gesunden Kontrollpersonen zu unterscheiden. Die Aussagekraft der FET Werte wurden dabei nicht signifikant durch die MCAD-spezifische Medikation beeinflusst. Der erweiterte FET Test könnte somit als diagnostische *in-vitro* Methode zur Abschätzung der Mastzellaktivität in MCAD Patienten eingesetzt werden und für den individuellen Patienten zur Therapieoptimierung beitragen.

## Background

The term “systemic mast cell activation disease” (MCAD) comprises disorders characterized by enhanced release of mediators from mast cells. To date, three classes of systemic MCAD have been classified, namely: *systemic mastocytosis* (SM), *idiopathic mast cell activation syndrome* (MCAS), and *mast cell leukemia* (MCL) [1-3]. SM is characterized by specific pathological mutations in exon 17 of the tyrosine kinase KIT (mainly KIT^D816V^) and mutation-dependent histological and immunohistochemical findings, as described in the World Health Organization (WHO) criteria for diagnosis of SM [4]. MCAS is diagnosed in patients presenting multiple symptoms, linked to mast cell-derived mediators, who do not fulfil the WHO criteria of SM [1-3,5-8]. MCL depicts an aggressive mast cell neoplasm, which is defined by an increased number of mast cells in bone marrow smears (≥20%) as well as numerous circulating mature mast cells in the peripheral blood (reviewed in [4]). Since MCL is extremely rare, this MCAD class was not included in the present explorative study.

Owing to known increased activity of mast cells in MCAD patients, detection of enhanced mediator release is part of the diagnostic algorithm of MCAD [1,2]. There are more than 200 different mediators which have been identified to be released by mast cells. However, only a few are being used as routine laboratory parameters in diagnosing MCAD, which include tryptase, histamine, and heparin (see e.g. Table 1 for levels of tryptase in the MCAD patients included in the present study). The pattern and extent of released mediators vary markedly in patients with MCAD, depending on several factors such as number and combination of mutated genes, the location of the activated mast cells in the body, and the type of trigger. Therefore, there is a need in additional, reliable, and meaningful biomarkers with less variability in diagnosing MCAD.

**Table 1:** **Characteristics of MCAD patients**

**MCAS**						**SM**					
**No.**	**Sex**	**Age**	Tryptase* [μg/L]	**KIT**	**Medication**	**No.**	**Sex**	**Age**	**Tryptase [μg/L]**	**KIT**	**Medication**
1	f	30	2.0	neg.	no	1	f	39	4.6	pos.	yes
2	f	36	3.3	neg.	no	2	f	44	3.3	pos.	yes
3	f	36	9.4	neg.	no	3	f	46	10.5	pos.	yes
4	f	45	3.4	neg.	no	4	f	53	27.0	pos.	yes
5	f	46	11.3	neg.	yes	5	f	55	4.9	pos.	yes
6	f	46	3.0	neg.	no	6	f	70	>200	pos.	yes
7	f	57	3.9	neg.	yes	7	m	44	24.7	pos.	yes
8	f	59	4.4	neg.	yes	8	m	51	>25	pos.	yes
9	f	61	3.6	neg.	no	9	m	52	40.7	pos.	yes
10	f	71	5.7	neg.	no	10	m	69	10.1	pos.	yes
11	m	20	8.1	neg.	no						
12	m	38	n.d.	neg.	no						
Mean		45.4				Mean		52.3			
Median		45.5				Median		51.5			
Range		[20–71]	[2.0-11.3]			Range		[39–70]	[3.3- > 200]		

f: female; m: male; n.d.: not determined; neg.: KIT^D816V^ negative; pos.: KIT^D816V^ positive; no: no MCAD-specific medication; yes: MCAD-specific medication (for details see Additional file 1); *ImmunoCAP^®^ Tryptase assay, normal range: < 11.4 μg/L.

Activated mast cells, amongst other mediators, rapidly generate eicosanoids such as prostaglandin D_2_ (PGD_2_), prostaglandin E_2_ (PGE_2_), and peptido-leukotrienes (pLTs) [9,10], which have been suggested to represent biomarkers of mast cell activation [11,12]. Determination of eicosanoid levels may thus represent a promising tool in diagnosing MCAD. However, quantification of these eicosanoids in serum or urine of MCAD patients is hampered by indeterminable and unpredictable individual factors which strongly influence their formation, release, spillover into blood or urine, and their degradation. These confounding factors in the analysis of eicosanoids present in blood and urine might be substantially reduced by applying a test, originally introduced as “*functional eicosanoid testing and typing”* (FET) in the diagnosis of aspirin-exacerbated respiratory disease (AERD) by Schäfer and colleagues [13]. The FET analysis detects and quantifies the interactions of both basal and triggered PGE_2_ and pLT release from peripheral blood leukocytes (PBLs) *in vitro* upon exposure to arachidonic acid (AA), acetylsalicylic acid (ASA), or substance P (SP). The measured eicosanoid levels are then processed by mathematical algorithms, which take into account known biochemical interactions of individual eicosanoids. The resulting FET value is therefore able to indicate and classify changes in eicosanoid balance (i.e. the complex pattern of formation, release and mutual interaction), reflecting some of the pathophysiologic mechanisms of the underlying diseases. In the clinical setting the standard FET was already successfully applied for detection, prognosis, and follow-up upon surgical/medical treatment of patients suffering from diseases like intrinsic asthma, nonsteroidal-anti-inflammatory (NSAID)-triggered hypersensitivity with and without nasal polyps and/or asthma, urticaria, inflammatory bowel disease (e.g. ulcerative colitis, Crohn’s disease), gastrointestinal cancer, as well as sepsis and systemic inflammatory response syndrome (for details, see [13] and further references therein).

Here we report the results of a promising explorative, diagnostic study, initiated to test the usefulness of FET analysis in diagnosing MCAD. Therefore, PBLs were collected from 22 MCAD patients and 20 healthy individuals and were analyzed by standard FET to try to distinguish patients with MCAD from healthy individuals. Additionally, basal, AA-, ASA-, and SP-triggered release of PGD_2_ from PBLs was included to potentially increase specificity, suitability, and relevance of FET analysis with respect to determination of mast cell activity in MCAD patients.

## Patients and methods

### Study population

Twenty-two Caucasian individuals, consecutively presenting with MCAD (12 with diagnosis of MCAS and 10 with diagnosis of SM, according to WHO criteria) from February to July 2012 at the St. Franziskus Hospital, Eitorf (Germany) or the Department of Oncology, Haematology and Palliative Care, District Hospital of Waldbröl (Germany), were randomly enrolled in the present prospective explorative study to better reflect disease diversity (for patients’ characteristics, see Table 1 and Additional file 1). The MCAD patients were clinically diagnosed according to the current criteria for MCAD [1-4]. Diagnostic criteria of SM and MCAS as well as the respective diagnostic findings of the individual patients are depicted in Additional file 2. The age of the patients ranged from 20 to 71 years (mean: 48.5 years; male to female ratio: 6:16). For comparison, twenty age-matched healthy individuals from Medical Clinic III, Friedrich-Alexander-University Erlangen-Nuremberg (Germany) were included (mean age: 44.3 years; range: 20–60 years; male to female ratio: 10:10). An extensive medical history of all healthy individuals was conducted. The presence of an dysfunctional immune system, immune defects, auto-immune disease, inflammatory disease of skin, airways, gastro-intestinal tract, atopy/allergy, hypersensitivity to drugs, aero- or food-allergens, food-additives, also exhaustive physical stress, immunization or xeno-immunoglobulin transfusion was ruled out. IgE-mediated allergy was ruled out by negative prick and specific IgE testing. Finally, no intake of antihistamines, steroids, or regular drugs other than oral contraceptives was accepted. To estimate the influence of MCAD-specific medication on eicosanoid patterns, subgroup analysis for MCAD patients without medication (MCAD-M; mean age: 40.3) and MCAD patients with MCAD-specific medication (MCAD+M; mean age: 52.7 years) were performed. In particular the majority of MCAS patients were devoid of MCAD-specific medication at the time of FET analysis, because they were included into the study shortly after making the diagnosis, but before starting MCAD-specific drug therapy. For diagnostic purposes, typical mast cell mediator-related symptoms were recorded by a standardized, validated questionnaire [14-16] (see also Additional file 1). Data on basal tryptase level, as analyzed by the “ImmunoCAP^®^ Tryptase” standard assay for quantification of tryptase in serum (Phadia GmbH, Freiburg, Germany; normal range < 11.4 μg/L) and KIT^D816V^ mutation status, determined by polymerase chain reaction based methods, were available for the included MCAD patients (see Table 1). For differential diagnosis, other conditions that could also be responsible for one or more of the reported symptoms were ruled out by unchanged relevant pathognomonic laboratory parameters, imaging techniques, and/or endoscopic methods. Before participating in the present study, all participants gave written informed consent to analysis of their data for scientific purposes and were free to withdraw from the study at any time. Blood samples were collected by standard routine diagnostic methods without further interventions. Therefore, a special ethical approval was not mandatory for the MCAD patients. Blood collection from healthy individuals, in general, was approved by the local ethical committee of the Friedrich-Alexander-University Erlangen-Nuremberg (Germany). The present study was performed in accordance with the ethical standards of the Helsinki Declaration 1975 (revised 2004).

### FET analysis and quantification of eicosanoids

Peripheral venous blood was collected from patients and healthy individuals and processed as described elsewhere [17-19]. Briefly, 4.5 ml of heparinized blood were used for isolation of PBLs. PBLs were resuspended in RPMI-1640 cell culture medium without fetal calf serum and adjusted to a cell density of 1 × 10^6^ cells/ml. PBLs were subsequently exposed to triggering factors, i.e. AA (10^−5^ M), ASA (10^−8^ M), SP (10^−12^ M), or diluent for 20 min. *In-vitro* formation and release of eicosanoids were stopped by freezing at -80 °C. Samples were then stored for up to four weeks at −80 °C before quantification of eicosanoids. After thawing and centrifugation, eicosanoids were quantitatively analyzed in the cell supernatants by specific enzyme immunoassays (EIA) using highly sensitive and specific competitive EIAs for PGE_2_, pLT (SIAT, Bad Essen, Germany), and PGD_2_ (Cayman, Ann Abor, USA) according to manufacturers’ protocols. Intra- and inter-assay variances for the PGE_2_, PGD_2_, and pLT EIA were 7.4% and 8.6%, < 20% and < 20.6%, and 5.6% and 8.1%, respectively. Recovery rate for PGE_2_, PGD_2_, and pLT was ≥ 95%; sensitivities of the EIAs were 3.0, 19.5, and 1.0 pg/ml, respectively ([20]; Cayman, Ann Abor, USA; [21]). The FET validation process demonstrated no interference of the test with ingestion of leukotriene receptor antagonists prior to blood sampling. The personnel performing the eicosanoid analyses were blinded to the source of the specimen (patient, healthy subject).

### Calculation of the FET value

The FET value represents the integrating endpoint of the standard FET assay. It considers both the metabolic network of the cyclooxygenase and 5-lipoxygenase pathways and summarizes the individual’s metabolic activities of triggered and un-triggered PBLs [13,19,22]. For calculation of the FET value, the raw data of released PGE_2_ and pLTs (analyzed in pg/ml) were processed according to a specific mathematical algorithm [19]. This is achieved by using both basal and triggered PGE_2_ and pLT levels, which are then integrated according to their biochemical interactions. The FET value is finally normalized, resulting in dimensionless categories between 0.0 and 3.0, which reflect the balance/imbalance (i.e. the complex pattern of formation, release and mutual interaction) of *in-vitro* PBL-derived PGE_2_ and pLT and thus constitute the patients’ individual eicosanoid pattern. Higher values are indicative of disturbance of the physiologic PGE_2_-pLT balance. Reliability of the calculated FET values was clinically confirmed for patients suffering e.g. from aspirin induced asthma and aspirin tolerant asthma (17, 22), gastric ulcer, intestinal cancer (23), NSAID-triggered hypersensitivity (18, 19), and urticaria (24).

### Calculation of supplemental PGD_2_ ratios

The supplemental PGD_2_ ratios R_A3_, R_S3_, and R_P3_ were calculated according to the following equations E1, E2, and E3:

E1 $$ {\mathrm{R}}_{\mathrm{A}3}={\mathrm{a}}_3/{\mathrm{b}}_3;\kern1.08em \to \kern0.97em \left(\mathrm{i}.\mathrm{e}.\ \mathrm{AA}\hbox{-} \mathrm{triggered}\ \mathrm{ratio}\right) $$

E2 $$ {\mathrm{R}}_{\mathrm{S}3}={\mathrm{s}}_3/{\mathrm{b}}_3;\kern1.09em \to \kern0.84em \left(\mathrm{i}.\mathrm{e}.\ \mathrm{ASA}\hbox{-} \mathrm{triggered}\ \mathrm{ratio}\right) $$

E3 $$ {\mathrm{R}}_{\mathrm{P}3}={\mathrm{p}}_3/{\mathrm{b}}_3;\kern1.09em \to \kern0.96em \left(\mathrm{i}.\mathrm{e}.\ \mathrm{SP}\hbox{-} \mathrm{triggered}\ \mathrm{ratio}\right) $$

with: 

a_3_: AA-triggered release of PGD_2_ [pg/ml];

b_3_: basal release of PGD_2_ [pg/ml];

s_3_: ASA-triggered release of PGD_2_ [pg/ml];

p_3_: SP-triggered release of PGD_2_ [pg/ml].

### Statistical methods

All data were tested for normal distribution using the Kolmogorov-Smirnov test with Lilliefors correction. If the assumption of normally distributed data could not be rejected, arithmetic group means ± standard deviations were calculated. The Student’s *t*-test for unpaired values was used for group comparisons. If the normality test failed in at least one of the study groups compared, all data were expressed as group medians with data ranges given in parentheses and comparison was calculated using the Mann–Whitney *U* test. *P* values were considered statistically significant, if *P* ≤ 0.05. These statistical analyses were performed using the SigmaPlot software (version 12.0, Systat Software GmbH, Erkrath, Germany). Optimal cut-off levels and misclassification rates were calculated by the method of ROC analysis, whereby the optimal cut-off point is derived by maximizing the Youden index [23]. Respective calculations were performed with the BIAS software for Windows (version 10.04; © 1989–2013 epsilon Verlag; http://www.bias-online.de).

## Results

### Clinical presentation of the MCAD patients

All patients demonstrated, to a varying degree, intermittently or chronically, a pattern of characteristic mast cell mediator-related symptoms (see Additional file 1) which was similar in patients with SM and MCAS. All SM patients were KIT^D816V^-positive and all MCAS patients were KIT^D816V^-negative. Five of 10 SM patients had tryptase blood levels > 20 μg/L, defined as the cut-off level in the corresponding WHO criteria. The remaining five SM patients and all MCAS patients exhibited normal basal tryptase levels (Table 1 and Additional files 1 and 2).

### Medication of the MCAD patients

All 10 patients with SM were on treatment with mast cell activity-reducing drugs (i.e. H_1_- and H_2_-antihistamines and cromoglicic acid) to alleviate symptoms induced by the various mast cell mediators, in particular by histamine (Table 1 and Additional file 1). Among the 12 patients with MCAS, only three were on medication with antihistamines, whereas eight did not take any medication, because they were enrolled in the study after making the diagnosis, but before starting symptomatic therapy. Two patients (one with MCAS, one with SM) ingested NSAIDs, another two patients with SM were treated with glucocorticoids, and three SM patients took the leukotriene receptor antagonist montelukast at the time of blood sampling. Nine of 22 patients (40.9%) did not receive any MCAD-specific medication (MCAD-M) at the time of blood sampling, whereas 13 of 22 patients (59.1%) were treated with MCAD-specific medication (MCAD+M). MCAD-specific as well as other regular medications of the enrolled MCAD patients varied highly, depending on the individual symptom constellation, intolerances, and overlapping pathologies.

### FET value

**Figure 1:**
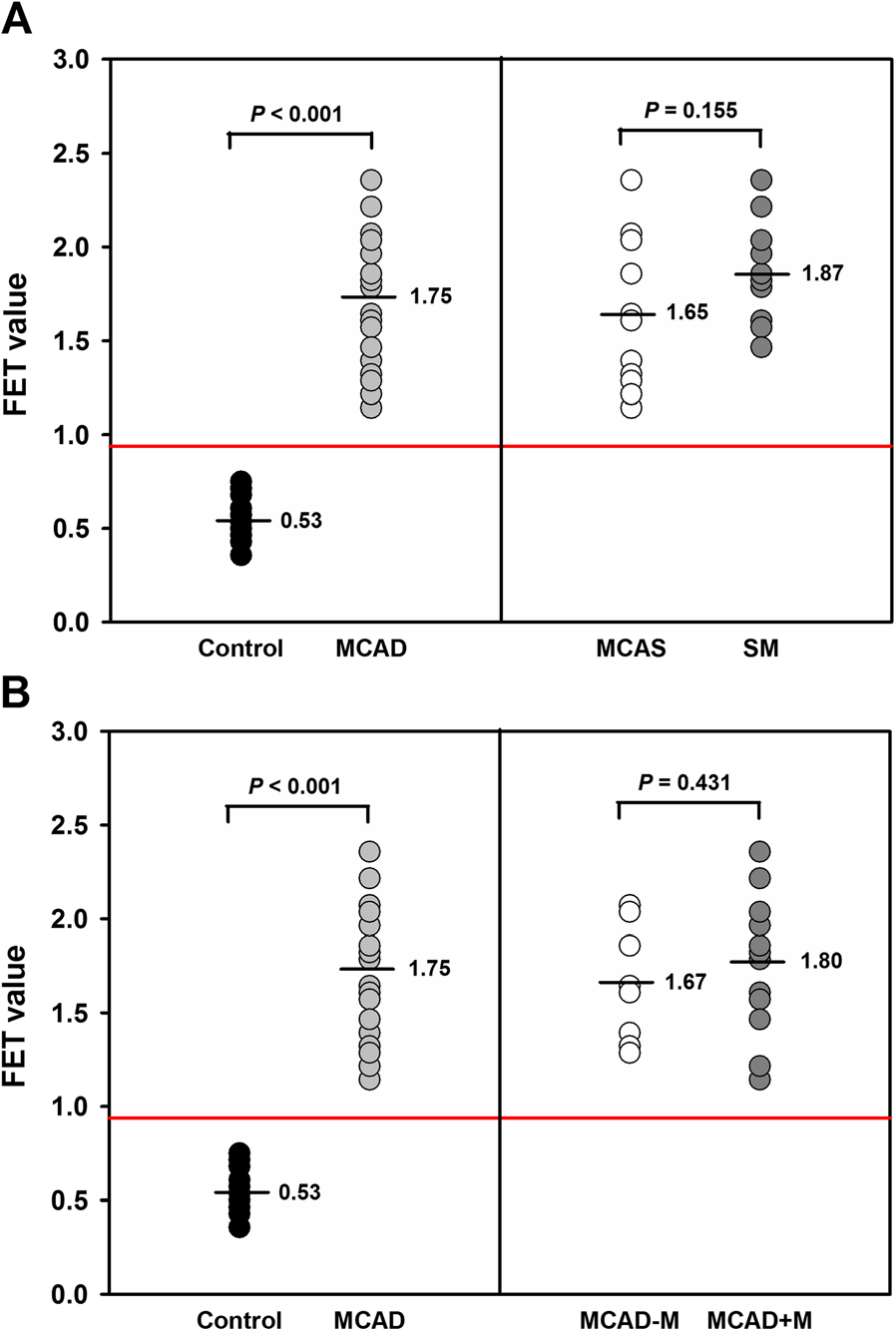
**FET values for patients suffering from MCAD.** Data represent the individual FET values of MCAD patients, derived group means (black horizontal lines), and the optimal cut-off value (red horizontal line) of 0.945 with 0.0% misclassification. Data were statistically analyzed using the Student’s *t*-test for unpaired values and ROC analysis, respectively. Control: healthy individuals (n = 20), MCAD: patients with mast cell activation disease (n = 22); **(A)** MCAS: MCAD patients with mast cell activation syndrome (n = 12); SM: MCAD patients with systemic mastocytosis (n = 10); **(B)** MCAD-M: MCAD patients without MCAD-specific medication (n = 9); MCAD + M: MCAD patients with MCAD-specific medication (n = 13).

The mean FET value (1.75 ± 0.356) of MCAD patients was significantly (*P* < 0.001) elevated, as compared to the healthy individuals (0.53 ± 0.119) (Figure 1). Hence, standard FET analysis distinguished MCAD patients from healthy individuals with a distinct cut-off FET value of 0.945 with 0.0% misclassification. A maximum FET value of 2.36 was detected for MCAS patient #7 and SM patient #1, whereas a minimum FET value of 1.14 was found for MCAS patient #5. SM patients exhibited a slightly higher mean FET value (1.87 ± 0.284) than MCAS patients (1.65 ± 0.389), but the difference did not reach statistical significance (*P* = 0.155; Figure 1A). This was similar for the mean FET value of MCAD patients without (MCAD-M: 1.67 ± 0.299) and with MCAD-specific drug treatment (MCAD+M: 1.80 ± 0.393; *P* = 0.43), indicating that the standard FET value, in the present explorative study, was suitable to distinguish MCAD patients from healthy individuals, even when patients were already treated with MCAD-specific drugs or NSAID (Figure 1B). To get an impression concerning robustness of the FET test and the FET algorithms with respect to NSAID intake raw data of MCAS patient #8, who ingested ASA prior to blood sampling, and SM patient #8, who indicated use of ibuprofen, were modeled with subsequent calculation of the FET value under the assumption that the NSAID medication led to inhibition of cyclooxygenase activity and thus underestimated prostaglandin values and, in addition, increase in 5-lipoxygenase activity with subsequent overestimation of pLT. However, even at the assumption of an unrealistically high under- (PGE_2_) and overestimation (pLT) of 50% none of the two patients would have been misdiagnosed by using the FET algorithms (see Additional file 3).

### Basal release of PGD_2_, PGE_2_, and pLT

**Figure 2:**
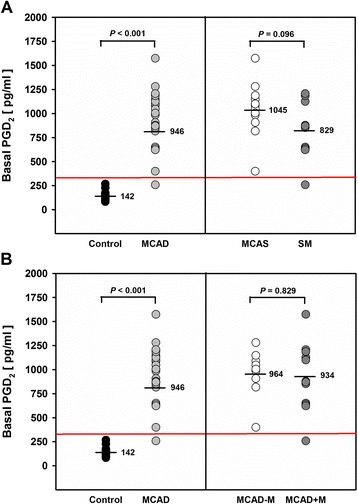
**Basal release of PGD**_**2**_ **from PBLs of MCAD patients.** Data represent individual PGD_2_ levels of MCAD patients and healthy controls, derived group means (black horizontal lines), and the optimal cut-off value (red horizontal line) of 333.31 pg/ml with 2.3% misclassification. Data were statistically analyzed using the Student’s *t*-test for unpaired values and ROC analysis, respectively. Control: healthy individuals (n = 20), MCAD: patients with mast cell activation disease (n = 22); **(A)** MCAS: MCAD patients with mast cell activation syndrome (n = 12); SM: MCAD patients with systemic mastocytosis (n = 10); **(B)** MCAD-M: MCAD patients without MCAD-specific medication (n = 9); MCAD + M: MCAD patients with MCAD-specific medication (n = 13).

We further analyzed the release of PGD_2_, because PGD_2_ represents a prominent mast cell eicosanoid, which is related to enhanced mast cell activity [10,11]. We thus hypothesized that integration of PGD_2_ into the FET analysis might reveal more MCAD-specific individual eicosanoid patterns. Mean basal release of PGD_2_ from PBLs was indeed markedly enhanced (6.6-fold) in patients with MCAD (946 ± 302.2 pg/ml; *P* < 0.001), as compared to healthy individuals (142 ± 47.8 pg/ml) (Figure 2A). A distinct cut-off value of 333.31 pg/ml was calculated, however, with a misclassification rate of 2.3%. PGD_2_ release from PBLs of MCAS patients (mean: 1045 ± 280.3 pg/ml) was higher than that from PBLs of SM patients (mean: 829 ± 298.0 pg/ml), but the difference did not reach statistical significance (*P* = 0.096) (Figure 2A). Maximum basal PGD_2_ level of 1574 pg/ml was observed for MCAS patient #5 whereas the lowest PGD_2_ level was observed for SM patient #1. MCAD-M and MCAD+M patients (mean: 964 ± 250.4 pg/ml and 934 ± 343.0 pg/ml, respectively) had similar mean basal PGD_2_ levels (Figure 2B), with a broader variance in the MCAD+M subgroup.

Mean basal release of PGE_2_ and pLT from PBLs of MCAD patients was significantly higher (1.8-fold, *P* = 0.005, and 3.2-fold, *P* < 0.001, respectively), than that of healthy controls (Additional file 4). However, basal PGE_2_ and pLT levels were less discriminative than the FET value (which considers both basal and triggered PGE_2_ and pLT levels and their biochemical interactions) or the basal PGD_2_ release concerning the discrimination of patients from healthy controls. ROC analysis indicated misclassification rates of 27.0% and 11.4% for basal PGE_2_ and pLT, respectively, when calculating the respective optimal cut-off levels of 207.65 pg/ml (PGE_2_) and 140.55 pg/ml (pLT). Notably, PBLs from MCAS patients released significantly more PGE_2_ (median: 228 pg/ml; range: 108–994 pg/ml; *P* = 0.018) than PBLs from SM patients (median: 136; range: 40–629 pg/ml) (Additional file 4). Mean basal PGE_2_ as well as pLT release was not significantly different in patients without and with MCAD-specific medication (data not shown).

On the basis of the marked basal release of PGD_2_ in MCAD-patients, we expanded PGD_2_ analysis by investigating AA-, ASA-, and SP-triggered release of PGD_2_, since these triggering factors are routinely used in standard FET analysis and are of relevance in MCAD.

### AA-triggered PGD_2_ release

AA, the physiological substrate of both the cyclooxygenase and 5-lipoxygenase pathways, seemed to be less active in stimulating PGD_2_ release from PBLs of MCAD patients than of healthy individuals, when expressed as PGD_2_ ratio (i.e. PGD_2_ level after AA stimulation divided by basal PGD_2_ release; Table 2): Median AA-triggered PGD_2_ ratio was significantly lower for MCAD patients (1.13; range: 0.20-3.26; including six non- or adverse-responder; *P* = 0.007) than for healthy controls (1.43; range: 0.88-2.46; one non-responder). There was no significant difference (*P* = 0.100) between MCAS (1.15; range: 0.63-1.63) and SM patients (1.07; range: 0.20-3.26); the drug treatment subgroups MCAD-M (1.17; range: 0.64-1.63) and MCAD+M (1.11; range: 0.20-3.26) also had similar median PGD_2_ ratios after AA stimulation (Table 2).

**Table 2:** **PGD**_**2**_ **ratios**

**Trigger**	**Control**	**MCAD**	**MCAS**	**SM**	**MCAD-M**	**MCAD + M**
	**Median ratio (range)**	**Median ratio (range)**	**Median ratio (range)**	**Median ratio (range)**	**Median ratio (range)**	**Median ratio (range)**
**AA**	1.43	1.13**	1.15**	1.07	1.17*	1.11*
	(0.88-2.46)	(0.20-3.26)	(0.63-1.63)	(0.20-3.26)	(0.63-1.63)	(0.20-3.26)
**ASA**	1.10	1.10	1.16	1.03	1.18	1.03
	(0.93-1.45)	(0.21-3.30)	(0.57-2.65)	(0.21-3.30)	(0.57-2.65)	(0.21-3.30)
**SP**	0.88	2.05***	1.71**	2.56***	2.07***	2.04***
	(0.40-1.34)	(0.79-6.64)	(0.79-4.87)	(1.61-6.64)	(0.99-4.87)	(0.79-6.64)

PGD_2_ ratio: PGD_2_ release from PBLs in the presence of a trigger, divided by basal PGD_2_ release. Control: healthy individuals (n = 10); MCAD: patients suffering from mast cell activation disease (n = 22); MCAS: MCAD patients with mast cell activation syndrome (n = 12); SM: MCAD patients with systemic mastocytosis (n = 10); MCAD-M: MCAD patients without MCAD-specific medication (n = 9); MCAD + M: MCAD patients with MCAD-specific medication (n = 13). Significantly different from control: ***P < 0.001, **P < 0.01, *P < 0.05, Mann–Whitney *U* test.

The observed lower median AA-triggered PGD_2_ ratio for MCAD patients was perhaps a result of the significantly higher basal PGD_2_ levels in this group, as in particular PBLs from some MCAD patients with basal PGD_2_ levels > 1000 pg/ml did not respond with enhanced, but with reduced release of PGD_2_ upon AA-triggering (Figure 2, Additional file 5). We thus hypothesized that high basal PGD_2_ release might limit further physiological AA-triggering. Nevertheless, median AA-triggered PGD_2_ level for MCAD patients were significantly higher (median: 964 pg/ml; *P* < 0.001) than for healthy controls (median: 180 pg/ml; range: 144–473 pg/ml), however with high variance (range: 236–1818 pg/ml). Because of the in part opposite effects of AA on PBLs from MCAD patients and the high range of dispersion of the AA-triggered PGD_2_ values, AA-triggered PGD_2_ release seemed not to be a sufficiently decisive endpoint for diagnostically differentiating MCAD patients from healthy controls.

### ASA-triggered PGD_2_ release

PBLs were exposed to ASA, a known trigger in some but not all MCAD patients. The median of the ASA-triggered PGD_2_ ratios (i.e. PGD_2_ level after incubation with ASA divided by basal PGD_2_ release) was not elevated in MCAD patients, when compared with healthy individuals (1.10 and 1.10, respectively; *P* = 1.000), irrespective of MCAD subclass or medication subgroup (for details see Table 2). However, when evaluating the individual ASA-triggered reactions on PGD_2_ release, highly variable effects were observed, in terms of no effect (e.g. SM patient #2), elevation (e.g. 3.3-fold increase for SM patient #1), or reduction in PGD_2_ release (e.g. decrease to 21% of the basal PGD_2_ level for SM patient #8). Variance of the ASA-triggered PGD_2_ ratio was markedly higher in MCAD patients (range: 0.21-3.30) than in healthy controls (range: 0.93-1.45) (Table 2).

**Figure 3:**
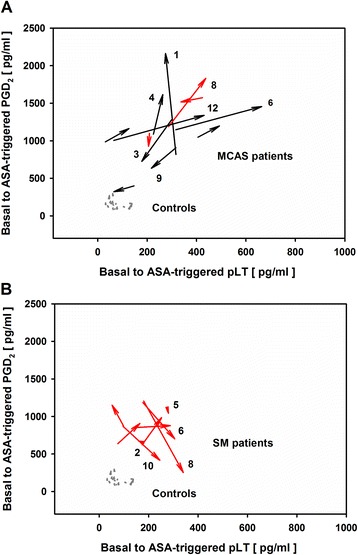
**Vector plots of ASA-triggered PGD**_**2**_ **and pLT dynamics derived from PBLs of MCAS and SM patients and healthy controls.** Origin of arrows: basal eicosanoid release; arrowheads: ASA-triggered eicosanoid release; grey arrows: healthy individuals (n = 20); black arrows: MCAD-M, MCAD patients without MCAD-specific medication (n = 9); red arrows: MCAD + M, MCAD patients with MCAD-specific medication (n = 12); black numbers: patients’ identification numbers (see Table 1). **(A)** MCAS: MCAD patients with mast cell activation syndrome (n = 12); **(B)** SM: MCAD patients with systemic mastocytosis (n = 9). One SM patient was not depicted in the figure, because of extraordinarily high pLT release, both basal and after ASA exposure (basal PGD_2_ and pLT release: 531 and 258 pg/ml, respectively; ASA-triggered PGD_2_ and pLT release: 2056 and 853 pg/ml, respectively).

Since ASA can be used therapeutically to decrease prostaglandin-related symptoms in MCAD patients [24,25], but can also trigger anaphylactic reactions, it was of special interest to look more closely into the individual ASA-triggered reactions of PBLs from MCAD patients. We, therefore, used vector plots to investigate individual ASA-triggered dynamics of the PGD_2_-pLT eicosanoid pattern, because both eicosanoids can account for profound adverse reactions. These plots summarize and integrate both basal (origin of arrows) and ASA-triggered PGD_2_ and pLT release (arrowhead) (Figures 3A and 3B). PBLs from healthy individuals were characterized by no or only marginal ASA-triggered modulation of the dynamics of eicosanoid release. In contrast, PBLs from MCAD patients demonstrated extended and highly individual responses to ASA with all kinds of different PGD_2_-pLT dynamics. PBLs from some patients reacted with a markedly increased release, of mainly PGD_2_ (e.g. MCAS patients #1 and #4, Figure 3A) or pLT [e.g. MCAS patients #6 (known to be ASA intolerant) and patient #12; Figure 3A] and some with an enhanced release of both PGD_2_ and pLT (e.g. MCAS patient #8, Figure 3A, and SM patient #1, not depicted in Figure 3B, because of extraordinarily high pLT values). In other MCAD patients no or only minor effects on the PGD_2_-pLT eicosanoid pattern [e.g. SM patients #2 (known to be ASA tolerant), and SM patient #5; Figure 3B] were observed upon *in-vitro* exposure to ASA. Furthermore, some patients demonstrated down regulation of PGD_2_ and/or pLT release (e.g. MCAS patients #3 and #9, Figure 3A, and SM patient #6, Figure 3B), in part with opposed effects on the other eicosanoid (e.g. SM patients #8 and #10, Figure 3B). Taken together, the effects of ASA on the PGD_2_-pLT dynamics in MCAD patients thus strongly differed between individuals with regard to the eicosanoid type/s involved as well as the extent and direction of the effect. Susceptibility of MCAD patients to ASA did not seem to be uniformly influenced by the individual MCAD-directed medication, because comparably treated patients did not demonstrate the same modulation pattern (see Additional file 6).

### SP-triggered PGD_2_ release

**Figure 4:**
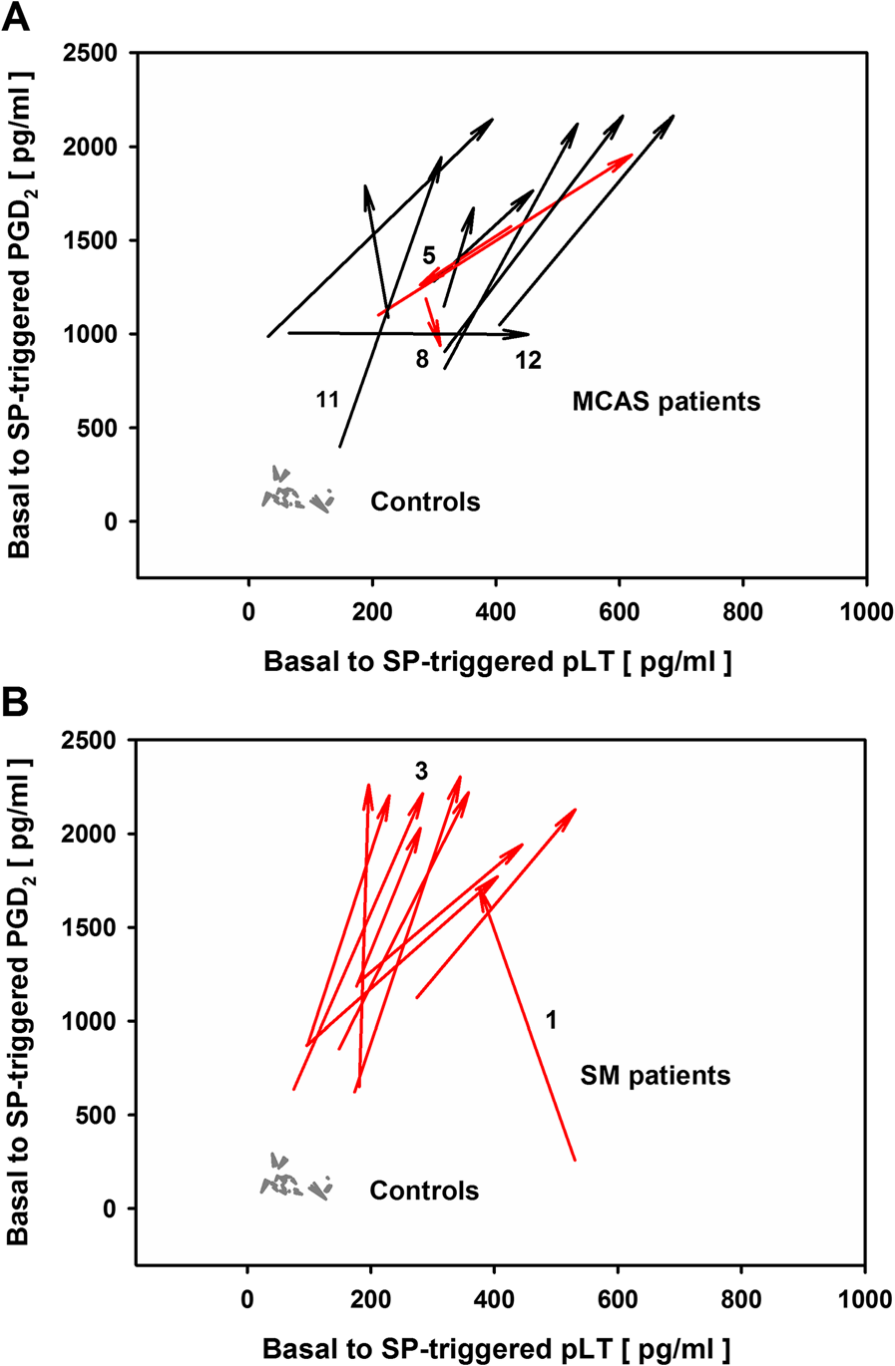
**Vector plots of SP-triggered PGD**_**2**_ **and pLT dynamics derived from PBLs of MCAS and SM patients and healthy controls.** Origin of arrows: basal eicosanoid release; arrowheads: SP-triggered eicosanoid release; grey arrows: control, healthy individuals (n = 20); black arrows: MCAD-M, MCAD patients without MCAD-specific medication (n = 9); red arrows: MCAD + M, MCAD patients with MCAD-specific medication (n = 13); black numbers: patients’ identification numbers (see Table 1) **(A)** MCAS: MCAD patients with mast cell activation syndrome (n = 12); **(B)** SM: MCAD patients with systemic mastocytosis (n = 10).

**Figure 5:**
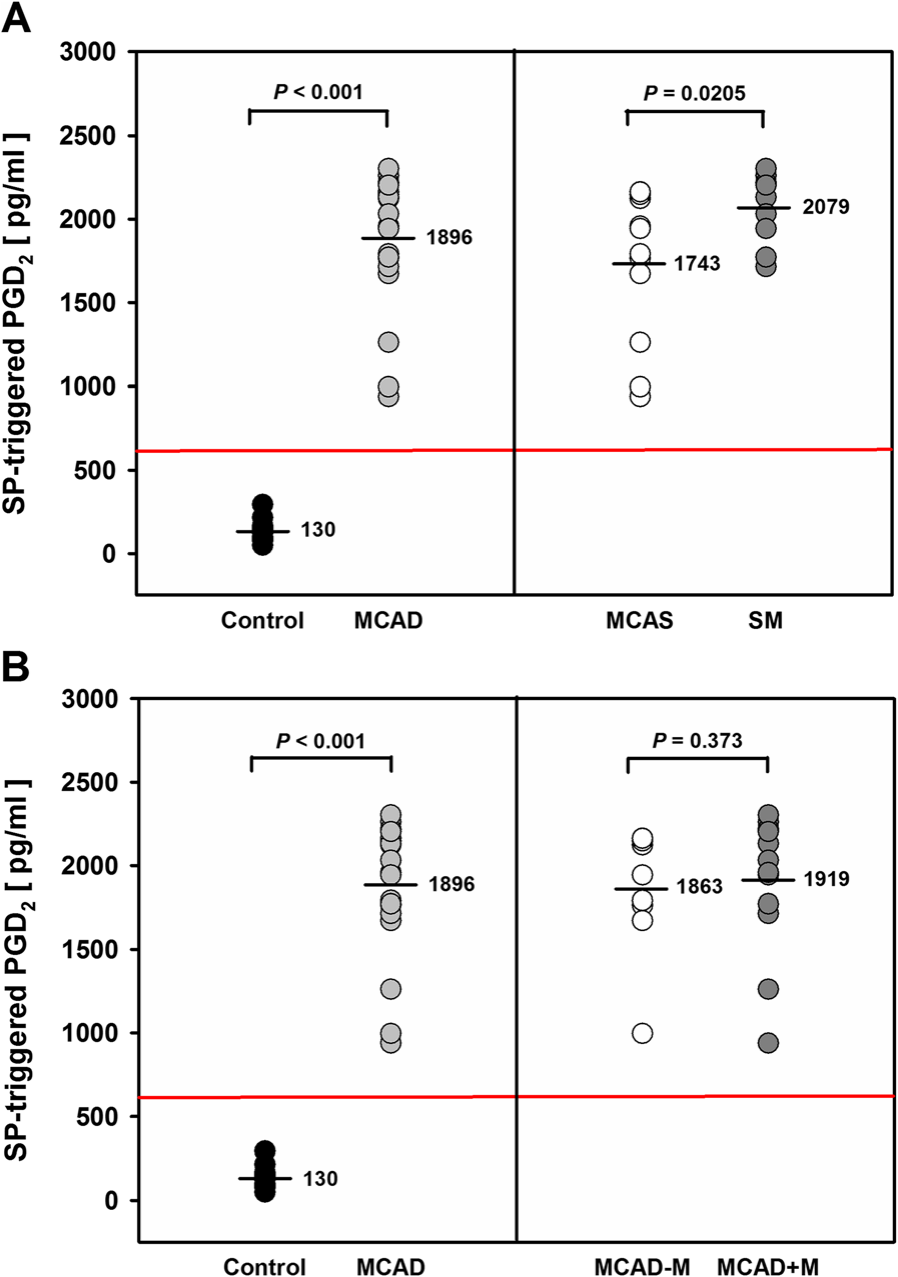
**SP-triggered release of PGD**_**2**_ **from PBLs of MCAD patients.** Data represent individual PGD_2_ levels of MCAD patients and healthy controls, derived group means (black horizontal lines), and the optimal cut-off value (red horizontal line) of 616.38 pg/ml with 0.0% misclassification. Data were statistically analyzed using the Student’s *t*-test for unpaired values and ROC analysis, respectively. Control: healthy individuals (n = 20), MCAD: patients with mast cell activation disease (n = 22); **(A)** MCAS: MCAD patients with mast cell activation syndrome (n = 12); SM: MCAD patients with systemic mastocytosis (n = 10); **(B)** MCAD-M: MCAD patients without MCAD-specific medication (n = 9); MCAD + M: MCAD patients with MCAD-specific medication (n = 13).

The neuropeptide SP was shown to be elevated in blood of patients suffering from mastocytosis [26]. In our study, SP caused a marked increase in PGD_2_ release from PBLs of 19 of 22 MCAD patients (Table 2, Figures 4 and 5). The SP-triggered PGD_2_ ratio (i.e. PGD_2_ level after SP stimulation divided by basal PGD_2_ level) was significantly higher in MCAD patients (median: 2.05; *P* < 0.001) than in healthy controls (median: 0.88), with wide ranges for both MCAD patients (0.79-6.64) and healthy controls (0.40-1.34) (Table 2). This was observed irrespective of the MCAD subclass or MCAD-specific drug treatment. The SP-triggered PGD_2_ ratio was significantly higher for SM patients (median: 2.56, range: 1.61-6.64; *P* = 0.044) than for MCAS patients (median: 1.71; range: 0.79-4.87). The medication subgroups demonstrated nearly identical SP-triggered PGD_2_ ratios (MCAD-M: median: 2.07; range: 0.99-4.87; MCAD+M: median 2.04; range: 0.79-6.64), however with a broader variance in MCAD-M patients. The SP-triggered PGD_2_ ratio was maximal for SM patient #1 (6.64), followed by MCAS patient #11 (4.87), and SM patient #3 (3.48). In 3 of 22 MCAD patients (MCAS patients #5, #8, and #12) no increase or even a slight decrease in SP-triggered PGD_2_-ratio of PBLs occurred.

In view of the obviously strong effect of SP on PGD_2_ release from PBLs of MCAD patients and considering the known biochemical interaction of the COX and 5-LOX pathways, we further analyzed the effect of SP on both PGD_2_ and pLT levels in more detail. Again, we used vector plots to investigate individual SP-triggered dynamics of the PGD_2_-pLT eicosanoid pattern. These plots summarize and integrate both basal (origin of arrows) and SP-triggered PGD_2_ and pLT release (arrowhead). PBLs from healthy individuals were characterized by no or only marginal SP-triggered modulation of the dynamics of eicosanoid release (Figures 4A and 4B). In contrast, PBLs from 19 out of 22 MCAD patients demonstrated marked increase in SP-triggered PGD_2_ release revealing a significantly (*P* < 0.001) enhanced mean PGD_2_ level of 1896 ± 389.7 pg/ml (range: 939 to 2303 pg/ml), as compared to 130 ± 53.5 pg/ml (range: 50–294 pg/ml) for healthy individuals (Figures 4 and 5). PBLs from SM patients exhibited significantly higher SP-triggered PGD_2_ release (2079 ± 207.2 pg/ml; *P* = 0.0205; Figure 5A) than PBLs from MCAS patients (1743 ± 446.0 pg/ml; Figure 5A). No significant difference was noted between the two medication groups MCAD-M and MCAD+M (1863 ± 376.8 and 1919 ± 411.9 pg/ml, respectively; *P* = 0.373; Figure 5B). Notably, two of the SP non-responders, concerning PGD_2_ release, instead showed enhanced pLT release upon SP-stimulation. Analysis of the vector plots demonstrated partially pronounced stimulation of SP-triggered pLT release for 18 of 22 MCAD patients (Figure 4). Mean pLT release amounted to 393 ± 137.2 pg/ml (range: 188–687 pg/ml) for MCAD patients, as compared to 72 ± 33.1 pg/ml (range: 23–131 pg/ml) for healthy individuals (Additional file 7). MCAS patients exhibited a higher mean pLT release of 433 ± 153.9 pg/ml than SM patients (mean: 345 ± 101.4 pg/ml) upon SP stimulation, but the difference did not reach statistical significance (*P* = 0.068; Additional file 7).

As depicted in Figures 4 and 5 and in Additional file 7, there was no overlap of the individual SP-triggered PGD_2_ and pLT levels of MCAD patients with that of healthy individuals. Subsequently, distinct cut-off values of 616.38 pg/ml and 159.59 pg/ml could be calculated for SP-triggered PGD_2_ and SP-triggered pLT levels, respectively, with each 0.0% misclassification rate. Especially the SP-triggered PGD_2_ level was highly decisive concerning distinction of MCAD patients from healthy controls (Figure 5).

## Discussion

MCAD is characterized by an enhanced release of mast cell-derived mediators, which are linked to a broad spectrum of mediator-related symptoms. Symptom patterns from MCAD patients are highly individual and can differ significantly from patient to patient, depending, amongst others, on the types and amounts of released mediators. Thus, confirmation of increased mediator release is mandatory in the diagnostic algorithm of MCAD [1,2]. Our present explorative study therefore investigated the significance of a PGD_2_-supplemented FET analysis with PBLs as a new *in-vitro* diagnostic tool in MCAD.

The functional *in-vitro* method of FET (i.e. “functional eicosanoid testing and typing”) was originally designed by Schäfer and colleagues for diagnosing aspirin-exacerbated respiratory disease (AERD; for review, see [13]). Accordingly, the standard version of FET analysis uses an AERD-adapted panel of eicosanoids (i.e. PGE_2_ and pLT), AERD-approved triggers (AA, ASA, SP), and also AERD-adapted mathematical algorithms for calculation of the FET value as the integrating test read out. The present study now applied for the first time standard FET analysis to evaluate PBL-derived eicosanoid patterns for MCAD patients. Irrespective of the AERD-focused methodological background of the FET approach, standard FET analysis clearly differentiated MCAD patients (both MCAS and SM patients) from healthy individuals, since markedly enhanced FET values were observed in the randomly recruited patient group. Data thereby enabled calculation of a distinctive cut-off value (i.e. 0.945) with a misclassification rate of 0.0%, indicating clear cut-off differences in the balance of PGE_2_ and pLT in MCAD patients and healthy individuals. Therefore, the standard FET analysis, which reflects the complex interactions of PGE_2_ and pLT [18] could be a useful *in-vitro* tool in distinguishing MCAD patients from healthy individuals.

The striking PGE_2_ and pLT imbalances we found in MCAD patients are in agreement with known rapid generation of both PGE_2_ and pLT by activated mast cells [9,10,27,28]. However, increased FET values have also been observed in other inflammatory diseases accompanied by pathological eicosanoid patterns, such as urticaria and asthma in patients suffering from NSAID-triggered hypersensitivity, gastroduodenal ulcer, and gastrointestinal cancer [13,29-33]. Those disorders are often found as MCAD-dependent and -independent comorbidities. Thus, standard FET analysis seems to lack MCAD specificity, but in combination with additional clinical findings, laboratory parameters, and/or imaging techniques, indicative for mast cell activation, it could be used to confirm the diagnosis of MCAD, irrespective of the biologic heterogeneity of the MCAD patients.

To yield more MCAD-specific eicosanoid patterns and to potentially enhance the power of FET analysis in diagnosing MCAD, standard FET analysis was supplemented with analysis of PBL-derived PGD_2_. PGD_2_ is the major prostanoid released by activated mast cells [34] and it was proposed to be an indicator of mast cell activity *in vivo*, because mast cells are known to produce the most significant quantities of PGD_2_ among leukocytes [11,12]. Furthermore, PGD_2_ is assumed to be involved in hemodynamic symptoms of systemic mast cell diseases and in other symptoms such as increased mucus secretion, bronchoconstriction, and pain [2,25,35,36].

In the present study, markedly higher basal release of PGD_2_ from PBLs of MCAD patients than from healthy controls was indeed demonstrated. This is in line with previous studies, which showed enhanced levels of the major PGD_2_ metabolite PGD-M in plasma and urine of patients with SM by modified mass-spectrometric methods [36-38]. Concerning cellular origin of PBL-derived PGD_2_, previous reports indicated that PBLs comprise hematopoietic mast cell-committed progenitor cells as well as small numbers of mature mast cells [39,40] and, in addition, small numbers of mature mast cells have been detected in peripheral blood cells of patients with SM [41]. Furthermore, CD34^-^KIT^+^ blood cells were detected that were identified to represent circulating precursor cells of mast cells, and their quantity was correlated with the severity of SM [42]. Therefore, markedly elevated PGD_2_ release from PBLs of MCAD patients, as measured by the PGD_2_-supplemented FET assay, might at least in part reflect mast cell activity, and PBLs might serve as suitable specimens for analyzing mast cell activity and individual eicosanoid release patterns of MCAD patients. But, other cell types such as T helper 2 cells [43], eosinophils, and basophils, primed by or in close cross-talk with activated mast cells could have also contributed to the high basal PGD_2_ release from PBLs of MCAD patients. For example, untriggered human blood eosinophils constitutively express hematopoietic prostaglandin D synthase and secrete PGD_2_ upon pre-incubation with AA [44]. Furthermore, IgE-mediated PGD_2_ release was documented for human basophils [45]. Notably, MCAD can be accompanied by eosinophilia and/or basophilia [46,47] and 4 of 22 MCAD patients in the present study showed eosinophilia or both eosinophilia and basophilia (Additional file 1). Thus, these cell types might also have contributed to PBL-derived PGD_2_ release. However, it was beyond the scope of our explorative study to evaluate in detail the cellular origin of PGD_2_ in the investigated samples. This has to be done in a respective future study.

Besides high PGD_2_ release, we also demonstrated enhanced mean basal release of PGE_2_ and pLT from PBLs of MCAD patients, as compared to healthy controls. This finding is in line with reports that mast cells are a major source of cysteinyl LTs and that urinary excretion of LTE_4_ is increased in patients suffering from SM [48,49]. Accordingly, increased release of both PGE_2_ and pLT may contribute to induction of various clinical symptoms in MCAD patients. Increased basal release of pLT from PBLs has also been observed in patients suffering from other diseases like AERD [13,33]. The disease-related elevated basal release of pLT in patients with AERD was shown to be due to increased activity of monocytes, granulocytes, and T-lymphocytes [17,19]. Similarly, detection of an elevated release of pLT from PBLs of MCAD patients in our study suggests that not only the activity of mast cells but also the activity of other immune competent cells may be increased in MCAD. This supports the idea that symptoms of MCAD, in particular in patients with high disease intensity, could be the consequence of an amplified cascade of basophilic, eosinophilic, or generalized leukocyte activation with subsequent eicosanoid and cytokine release. Induction of eicosanoid and cytokine release from these cell types might thereby be triggered by liberation of certain mast cell-derived mediators [50] and pLTs are in turn able to enhance degranulation of mast cells through paracrine and autocrine mechanisms [51,52].

Quantification of basal release of PGD_2_, PGE_2_, and pLT from PBLs of MCAD patients by FET analysis may not only aid in indicating mast cell activity or leukocyte activity in general, but might also support therapeutic decision processes for individual treatment of MCAD patients. In this respect, our results point to therapeutic options in MCAD (in addition to antihistamines and mast cell stabilizing drugs), such as the use of anti-LT drugs (i.e. 5-lipoxygenase inhibitors or LT-receptor antagonists) in patients demonstrating high basal and/or triggered pLT levels. Notably, the beneficial use of the leukotriene receptor antagonist montelukast has already been reported in the treatment of systemic and cutaneous mastocytosis [53-55]. Besides its function as leukotriene receptor antagonist montelukast was, in addition, shown to exhibit some 5-LOX-inhibiting potential [56,57], which might further aid in controlling pLT release in MCAD patients. In patients with high basal and/or triggered levels of PGE_2_ and/or PGD_2_ in FET analysis prescription of cyclooxygenase inhibitors such as ASA or coxibs could in turn be considered, as these patients might benefit from COX-inhibition. Further studies are necessary to evaluate whether the present *in-vitro* method is suited to support such therapeutic decision processes.

NSAIDs such as ASA, which can be beneficial for some MCAD patients by reducing prostanoid-related symptoms [24,25], can also be a strong trigger in some MCAD patients, causing sometimes life-threatening anaphylactic reactions [58]. Thus, prior knowledge of susceptibility of MCAD patients to ASA is important. In the present study, we observed highly variable effects of ASA on *in vitro*-treated PBLs from MCAD patients in terms of no effect, elevation, and reduction of PBL-derived PGD_2_ and pLT release, with both same and opposing effects. This clearly contrasts with the almost homogenous SP-triggered effects on PBLs from MCAD patients and may reflect the highly individual susceptibility of MCAD patients to ASA. For instance, one patient with known ASA intolerance exhibited significant induction of PGD_2_ and pLT release by ASA, whereas another patient with known tolerance demonstrated no effect of ASA on eicosanoid production. We thus assume that the new functional *in-vitro* FET approach has the potential to detect free of risk the individual predisposition for adverse or tolerant reactions of MCAD patients to diverse drug treatment. As, in the present study, susceptibility of the individual patients to ASA (tolerance, intolerance) was not recorded explicitly at the time of blood sampling, supporting data are limited. Thus, to further evaluate this assumption it needs future validation studies with larger groups of MCAS and SM patients, appropriate reference groups, and extended anamnestic endpoints.

As described above, FET analysis considers both basal and triggered eicosanoid release from PBLs to receive additional information on the dynamics of eicosanoid release and on respective individual eicosanoid patterns. Besides ASA, PBLs were also exposed to the neuropeptide SP. SP is a strong trigger for mast cells of different origin [26,27,59] and is known to cause *de-novo* synthesis of PGD_2_ and leukotrienes [60]. Notably, elevated plasma levels of SP and other neuropeptides have been detected in patients with SM, thereby correlating with mast cell load [26] and urinary levels of SP were increased in some patients with urticaria pigmentosa [61]. Thus, SP seems to be a relevant endogenous trigger of mast cell activity in MCAD. In our explorative diagnostic study, the release of PBL-derived PGD_2_ was markedly elevated in 86% of the MCAD patients upon *in-vitro* exposure of cells to SP. Such an effect was absent in the healthy control group. SP-triggered PGD_2_ release from PBLs alone or in combination with SP-triggered pLT release offered the most decisive discrimination of MCAD patients from healthy individuals, as the SP-triggered PGD_2_ levels of all MCAD patients were higher than that of the healthy individuals, with a distinct cut-off value for SP-triggered PGD_2_ of 616 pg/ml.

The finding that PBLs of healthy controls exhibited nearly no SP-triggered effects *in vitro*, suggests that PBLs of MCAD patients are more prone to SP stimulation. Enhanced SP sensitivity has previously been demonstrated for urticaria pigmentosa skin mast cells. SP challenge mediated higher spontaneous histamine release from urticarial pigmentosa mast cells than from mast cells of healthy skin [62]. Enhanced SP sensitivity may thereby be due to increased expression of SP receptors on pathologically altered mast cells, as some mast cell cell lines were shown to express SP receptors [63,64]. Additionally, van der Kleij and co-workers [65] demonstrated induction of neurokinin 1 receptor expression in normal bone marrow-derived mast cells by interleukin 4 and the tyrosine kinase KIT ligand stem cell factor, and thus KIT activation appears to make the cells more sensitive to SP [60,66]. Since KIT is altered and constitutively active in SM patients, stem cell factor independent KIT activity might also lead to enhanced neurokinin 1 receptor expression with subsequently increased SP sensitivity. But, also neurokinin 1 receptor-independent SP-mediated mechanisms of mast cell activation might occur [65]. Notably, at lower SP levels, SP-triggered PGD_2_ and pLT release seemed to take place in the absence of mast cell degranulation [60]. Nevertheless, the mechanisms behind increased SP-sensitivity of PBLs from MCAD patients remain to be further elucidated.

The increased susceptibility of PBLs from MCAD to SP and especially SM patients, as found in the present study, together with the documented elevated blood levels of neuropeptides in MCAD patients [24,61] may support the concept of SP-mediated enhanced release of cytokines from PBLs. These cytokines could in turn induce symptoms by activating mast cells [67] and/or acting upon other effector cells. Thus, to reduce mast cell activity and MCAD-associated increased PBL activity neuropeptide receptor antagonists, acting on the neurokinin-1 receptor for SP or on the Mas-related gene receptor X2, as a common receptor for SP, somatostatin, and VIP [65,68], might be additional promising treatment options in the therapy of MCAD.

The PGD_2_-supplemented FET approach constitutes a remarkable improvement in determining increased mediator release in MCAD patients, compared to e.g. PGD-M quantification in plasma and urine by mass-spectrometric methods or quantification of serum tryptase levels. First of all, the new functional diagnostic test offers evaluation of individual eicosanoid patterns. It thereby integrates multiple mediators, known mediator interactions, and also basal and triggered eicosanoid dynamics. As a functional *ex-vivo* approach, this assay is less susceptible to unpredictable individual factors influencing formation, release, and spill over into blood or urine of mediators, or their degradation. Interestingly, determination of both basal mediator release and mediator levels during or shortly after a triggering “event” with assumed mast cell activation has recently been proposed as one criterion for diagnosing MCAS [2,8]. But, this does not seem easy to perform *in vivo*, because of the unpredictability of “events” and the in part unstable mediators or metabolites. In this context, the “event”-independent PGD_2_-supplemented FET might be a very useful tool, because it can simulate “events” *ex vivo* with isolated PBLs of MCAD patients. Finally, in contrast to e.g. mass-spectrometric methods, the presented FET approach for diagnosing MCAD is easier to perform, more affordable, and suitable for widespread use in clinical laboratories.

## Conclusions

Taken together, we demonstrate that the PGD_2_-supplemented FET assay represents a suitable *in-vitro* tool in the diagnostics of MCAD and for monitoring leukocyte and more generally disease activity. The standard FET value can unambiguously differentiate MCAD patients from healthy individuals, irrespective of patients’ individual medication or MCAD subclass. Our findings also show that PGD_2_ analysis may provide some specificity to FET analysis for its use in the diagnostic process of MCAD. However, for a more important and decisive role of the test in the diagnostic work-up of MCAD, the ability of the PGD_2_-supplemented FET to discriminate MCAD patients from patients with immunological/allergic and/or other inflammatory diseases will have to be evaluated in well-designed future studies. In particular, SP-triggered release of PGD_2_ and pLT from PBLs seems to be promising estimators of metabolic hyperactivity of PBLs from MCAD patients and may aid in distinguishing MCAD patients from healthy subjects and perhaps other diseases. Additionally, FET data may provide some predictive hints whether cyclooxygenase inhibitors or receptor antagonists are appropriate options for the individual patient. The PGD_2_-supplemented FET assay may serve as a new cost-effective and robust diagnostic *in-vitro* tool in diagnosing and monitoring MCAD and deserves further evaluation and validation with development of MCAD-specific algorithms.

## Additional files

Additional file 1:
**Characteristics and symptoms of MCAD patients.**
Additional file 2:
**Fulfilled diagnostic criteria of MCAD patients enrolled in the present study.**
Additional file 3:
**Modeling of FET values for NSAID-treated patients.**
Additional file 4:
**Basal release of PGE**_**2**_ **and pLT from PBLs of MCAD patients.**
Additional file 5:
**Vector plots of AA-triggered PGD**_**2**_ **and pLT dynamics derived from PBLs of MCAS and SM patients and healthy controls.**
Additional file 6:
**Different medications and stimulation by arachidonic acid (AA), acetylsalicylic acid (ASA), and substance P (SP) of PGE**_**2**_ **, pLT and PGD**_**2**_ **release.**
Additional file 7:
**SP-triggered release of pLT from PBLs of MCAD patients.**

## Abbreviations

AA: Arachidonic acid
AERD: Aspirin-exacerbated respiratory disease
ASA: Acetylsalicylic acid
FET: Functional eicosanoid testing and typing
MCAD: Systemic mast cell activation disease
MCAS: Idiopathic mast cell activation syndrome
MCL: Mast cell leukaemia
PBLs: Peripheral blood leukocytes
NSAID: Nonsteroidal-anti-inflammatory drug
pLTs: Peptido-leukotrienes
PGE_2_: Prostaglandin E_2_
PGD_2_: Prostaglandin D_2_
SM: Systemic mastocytosis
SP: Substance P
