# Supplementary material for: Prostaglandin D2-supplemented “functional eicosanoid testing and typing” assay with peripheral blood leukocytes as a new tool in the diagnosis of systemic mast cell activation disease: an explorative diagnostic study
Source: J Transl Med. 2014 Aug 12;12:213. doi: 10.1186/s12967-014-0213-2 (PMC4283146; doi:10.1186/s12967-014-0213-2)
Supplement: Additional file 2: — Fulfilled diagnostic criteria of MCAD patients enrolled in the present study. [file 12967_2014_213_MOESM2_ESM.pdf]

Provisional international criteria to define *idiopathic mast cell activation syndrome* (*MCAS patients*) **:**

Patient ID	1	2	3	4	5	6	7	8	9	10	11	12
**Major criteria**												
• Multifocal or disseminated increase in number of mast cells in bone marrow biopsies and/or in sections of other extracutaneous organ(s) (e.g., gastrointestinal tract biopsies; CD117-, tryptase- and CD25-stained)	✓		✓	✓		✓	✓	✓	✓	✓	✓	
• Unique constellation of clinical complaints as a result of a pathologically increased mast cell activity (mast cell mediator release syndrome)	✓	✓	✓	✓	✓	✓	✓	✓	✓	✓	✓	✓
**Minor criteria**												
• Mast cells in bone marrow or other extracutaneous organ(s) show an abnormal morphology (>25%) in bone marrow smears or in histologies												
• Mast cells in bone marrow express CD2 and/or CD25												
• Detection of genetic changes in mast cells from blood, bone marrow or extracutaneous organs for which an impact on the state of activity of affected mast cells in terms of an increased activity has been proved.												
• Evidence of a pathologically increased release of mast cell mediators by determination of the content of :												
○ Tryptase in blood: basically increased or increase in serum total tryptase of 20% above baseline plus 2 ng/mL during or within 4 h after a symptomatic period					✓							
○ Heparin in blood				✓					✓		✓	✓
○ N-methylhistamine in urine				✓								
○ Chromogranin A in blood								✓				
○ Other mast cell-specific mediators (e.g., leukotrienes, prostaglandin D_2)	✓	✓	✓	✓	✓	✓	✓	✓	✓	✓	✓	✓
• Response of symptoms to histamine H1 and/or H2 receptor antagonists or other mast cell-targeting agents such as cromoglicic acid		✓			✓			✓				

Diagnosis of MCAS is given if both of the two major criteria or the second major criterion and at least one mino criterion is fulfilled.

✓ = Diagnostic criterion fulfilled

Patient 2: Prescription of medication after blood sampling for the explorative study with subsequent feedback. Therefore no medication was indicated for this patient in Table 1 of the explorative study.

WHO criteria to define *systemic mastocytosis* (*SM patients*) :

**Patient ID**	**1**	**2**	**3**	**4**	**5**	**6**	**7**	**8**	**9**	**10**
**Major criterion**										
• Multifocal, dense aggregates of mast cells (15 or more cells) in bone marrow biopsies and/or biospies of other extracutaneous tissues and confirmed by tryptase immunohistochemistry or other special stains	✓	✓	✓	✓		✓	✓	✓	✓	✓
**Minor criteria**										
• Atypical or spindle shaped appearance of at least 25% of the mast cells in the diagnostic biopsy			✓		✓	✓	✓			
• Expression of CD2 and/or CD25 by mast cells in bone marrow, blood, or extracutaneous organs			✓		✓	✓	✓			
• KIT codon 816 mutation in bone marrow, blood or extracutaneous organs	✓	✓	✓	✓	✓	✓	✓	✓	✓	✓
• Persistent elevation of serum total tryptase levels > 20 ng/ml				✓			✓	✓	✓	

Diagnosis of SM is given if the major criterion and one minor criterion are fulfilled or if at least 3 minor criteria apply.

✓ = Diagnostic criterion fulfilled
