# Supplementary material for: Prostaglandin D2-supplemented “functional eicosanoid testing and typing” assay with peripheral blood leukocytes as a new tool in the diagnosis of systemic mast cell activation disease: an explorative diagnostic study
Source: J Transl Med. 2014 Aug 12;12:213. doi: 10.1186/s12967-014-0213-2 (PMC4283146; doi:10.1186/s12967-014-0213-2)
Supplement: Additional file 3: — Modeling of FET values for NSAID-treated patients. [file 12967_2014_213_MOESM3_ESM.pdf]

Modeling of FET values for NSAID-treated patients

Assumed inhibition of *in vitro* PGE_2 release and increase in pLT release due to NSAID ingestion of patients [%]		**0**	**10**	**30**	**50**
**FET value**					
**MCAS patient No. 8:**	**effecting all eicosanoids**	**1.214**	**1.214**	**1.214**	**1.357**
	effecting only basal eicosanoids	**1.214**	1.214	1.679	1.786
	effecting only AA-triggered eicosanoids	**1.214**	1.321	1.357	1.393
	effecting only ASA-triggered eicosanoids	**1.214**	1.036	0.949	1.036
	effecting only SP-triggered eicosanoids	**1.214**	1.214	1.286	1.429
**FET value**					
**SM patient No.8:**	**effecting all eicosanoids**	**1.964**	**1.750**	**1.464**	**1.321**
	effecting only basal eicosanoids	**1.964**	1.786	1.929	1.929
	effecting only AA-triggered eicosanoids	**1.964**	1.893	1.857	1.964
	effecting only ASA-triggered eicosanoids	**1.964**	1.643	1.321	1.357
	effecting only SP-triggered eicosanoids	**1.964**	1.821	2.036	2.036

The standard FET values were calculated for MCAS patient No. 8 (ASA ingestion) and SM patient No. 8 (ibuprofen ingestion) according to the normal FET algorithms also used in the present study. By assuming no effect of NSAID ingestion on the *in vitro* eicosanoid release, the measured raw data were used as default data for the FET calculation. The subsequent FET values thus correspond to the regularly quantified standard FET value for these patients (here represented by 0% inhibition), as given in Figure 1. Theoretical 10, 30, and 50% inhibition (PGE_2) and induction (pLT) effects by ingestion of NSAID were modeled for either affecting only a single parameter or all parameters which are considered in the FET algorithm by modifying the default data accordingly with subsequent FET calculation.

Depending on the assumed interferences with eicosanoid metabolism, based on a simplified biochemical interactions of eicosanoids, as explained previously, potential misclassification was evaluated for both patients by calculating the respective FET values. In more detail: For MCAS patient No. 8 the standard FET value as given in Figure 1 may represent a maximal overestimation of 21.8% or underestimation of 47.1% depending on the specific assumptions. For SM patient No. 8 the standard FET value as given in Figure 1 may represent a maximal overestimation of 3.7% or underestimation of 32.7%. By looking on the calculated cut-off value for the standard FET in the present study (i.e. 0.945), none of the two patients with NSAID ingestion would have been misclassified or not been detetced by the standard FET *in vitro* approach when considering potential over- or underestimations due to NSAID ingestion.
