# Supplementary material for: Prostaglandin D2-supplemented “functional eicosanoid testing and typing” assay with peripheral blood leukocytes as a new tool in the diagnosis of systemic mast cell activation disease: an explorative diagnostic study
Source: J Transl Med. 2014 Aug 12;12:213. doi: 10.1186/s12967-014-0213-2 (PMC4283146; doi:10.1186/s12967-014-0213-2)
Supplement: Additional file 4: — Basal release of PGE 2 and pLT from PBLs of MCAD patients. [file 12967_2014_213_MOESM4_ESM.pdf]

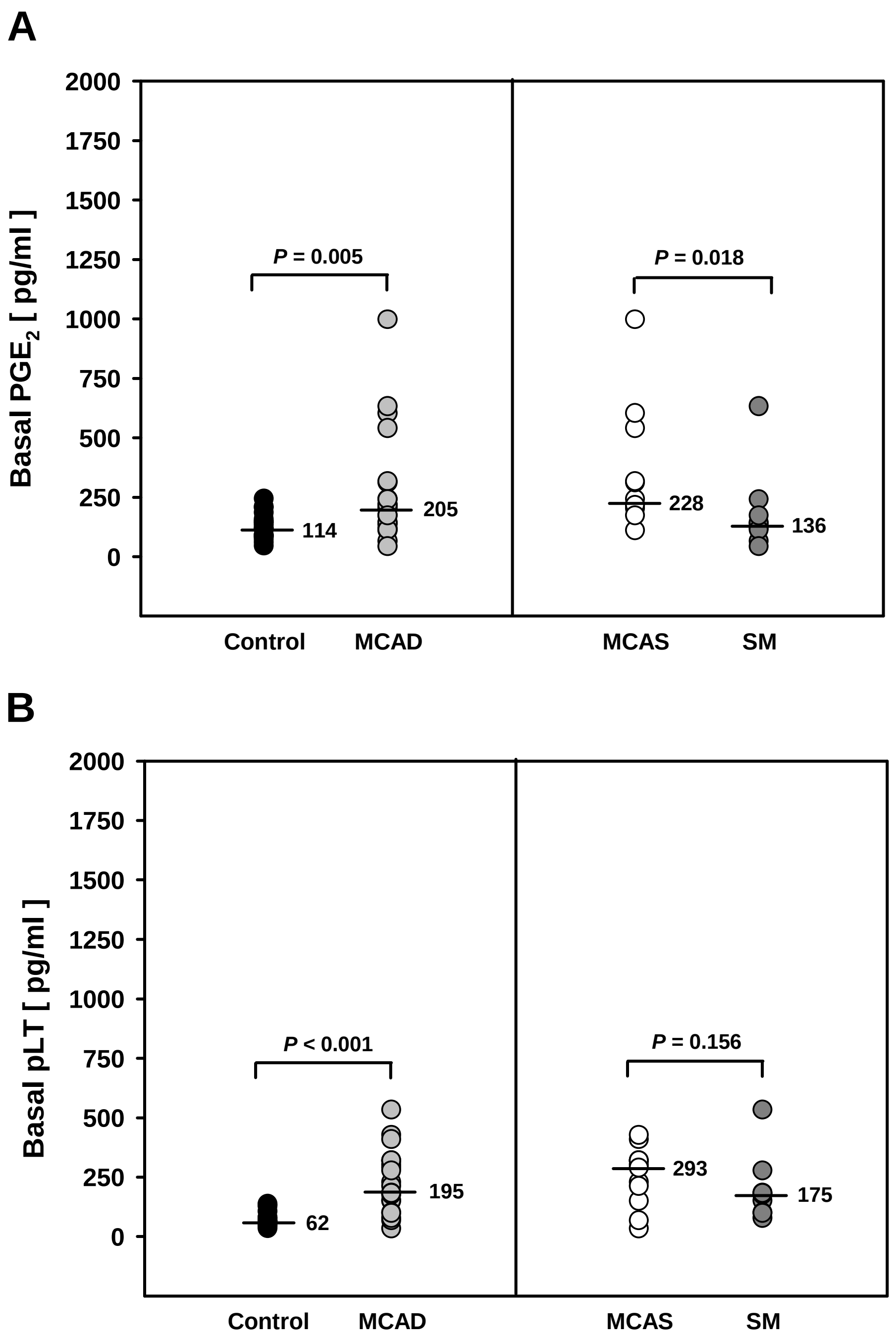

Additional file 4: **Basal release of PGE_2 and pLT from PBLs of MCAD patients.** Data represent (**A**) individual PGE_2 levels and (**B**) individual pLT levels of MCAD patients and healthy controls and the derived group medians (black horizontal lines). Data were statistically analysed using the Mann-Whitney *U* test, because of in part not normally distributed data. Control: healthy individuals (n = 20), MCAD: patients with mast cell activation disease (n = 22); MCAS: MCAD patients with mast cell activation syndrome (n = 12); SM: MCAD patients with systemic mastocytosis (n = 10).
