# Supplementary material for: Prostaglandin D2-supplemented “functional eicosanoid testing and typing” assay with peripheral blood leukocytes as a new tool in the diagnosis of systemic mast cell activation disease: an explorative diagnostic study
Source: J Transl Med. 2014 Aug 12;12:213. doi: 10.1186/s12967-014-0213-2 (PMC4283146; doi:10.1186/s12967-014-0213-2)
Supplement: Additional file 5: — Vector plots of AA-triggered PGD 2 and pLT dynamics derived from PBLs of MCAS and SM patients and healthy controls. [file 12967_2014_213_MOESM5_ESM.pdf]

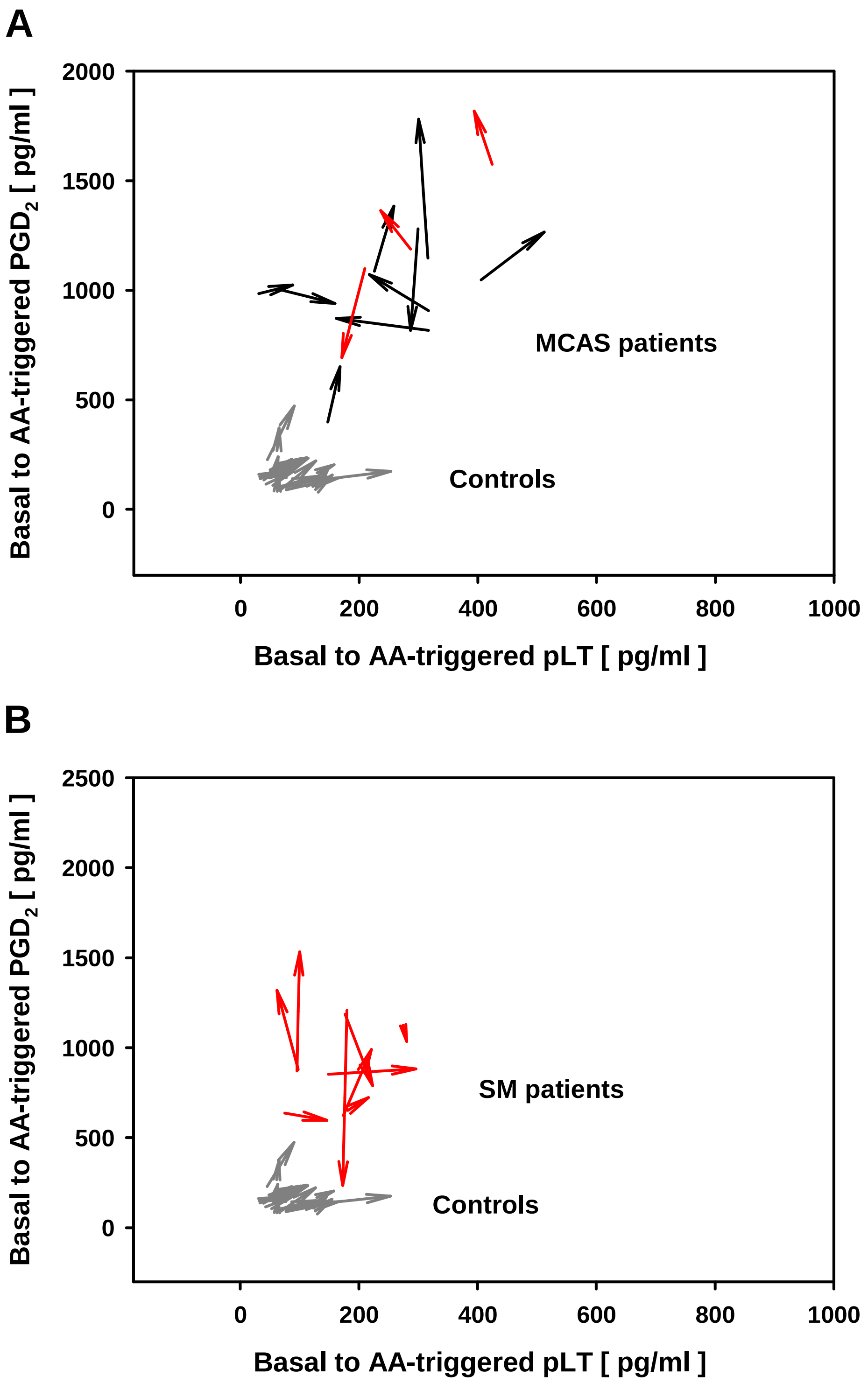

Additional file 5: Vector plots of AA-triggered PGD_2 and pLT dynamics derived from PBLs of MCAS and SM patients and healthy controls. Origin of arrows: basal eicosanoid release; arrowheads: AA-triggered eicosanoid release; grey arrows: control, healthy individuals (n = 20); black arrows: MCAD-M, MCAD patients without MCAD-specific medication (n = 9); red arrows: MCAD+M, MCAD patients with MCAD-specific medication (n = 12). (**A**) MCAS: MCAD patients with mast cell activation syndrome (n = 12); (**B**) SM: MCAD patients with systemic mastocytosis (n = 9). One SM patient was not depicted in the figure, because of extraordinarily high pLT release, both basal and after AA exposure (basal PGD_2 and pLT release: 531 and 258 pg/ml, respectively; AA-triggered PGD_2 and pLT release: 1810 and 843 pg/ml, respectively).
