# Supplementary material for: Prostaglandin D2-supplemented “functional eicosanoid testing and typing” assay with peripheral blood leukocytes as a new tool in the diagnosis of systemic mast cell activation disease: an explorative diagnostic study
Source: J Transl Med. 2014 Aug 12;12:213. doi: 10.1186/s12967-014-0213-2 (PMC4283146; doi:10.1186/s12967-014-0213-2)
Supplement: Additional file 6: — Different medications and stimulation by arachidonic acid (AA), acetylsalicylic acid (ASA), and substance P (SP) of PGE 2 , pLT and PGD 2 release. [file 12967_2014_213_MOESM6_ESM.pdf]

Additional file 6: Different medications and stimulation by arachidonic acid (AA), acetylsalicylic acid (ASA), and substance P (SP) of PGE_2, pLT and PGD_2 release.

Arachidonic acid (AA) stimulation, qualitative evaluation:

Medication & Patient	Eicosanoid		
	PGE_2	pLT	PGD_2
**H1-RA**			
MCAS 7	↓	→	↓
SM 5	→	→	→
**H1-RA + ASA**			
MCAS 8	→	↓	↑
**H1-RA + DNCG + H2-RA**			
MCAS 5	↑	→	↑
SM 4	↑	↑	→
SM 3	↓	↑	→
**H1-RA + DNCG + H2-RA + LT-RA**			
SM 2	→	↑	↑
SM 6	→	→	↓
SM 1 (- DNCG)	↑	↑	↑
**H1-RA + DNCG + H2-RA + Ibuprofen**			
SM 8	↑	→	↓
**Prednisolone**			
SM 7	↑	↑	↑
SM 10	↑	→	↑

MCAS: mast cell activation disease; SM: systemic mastocytosis; H1-RA: H1-receptor antagonist; H2-RA: H2-receptor antagonist; AA: arachidonic acid; DNCG: cromoglicic acid disodium salt: LT-RA: leukotriene-receptor antagonist; ↑ : increase, ↓ : decrease, or → : no significant effect after stimulation with AA, as compared to basal release.

Acetylsalicylic acid (ASA), qualitative evaluation:

Medication & Patient	Eicosanoid		
	PGE_2	pLT	PGD_2
**H1-RA**			
MCAS 7	↓	→	↓
SM 5	↓	↑	→
**H1-RA + ASA**			
MCAS 8	↑	↑	↑
**H1-RA + DNCG + H2-RA**			
MCAS 5	↓	→	↑
SM 4	↑	↑	→
SM 3	↓	↑	↑
**H1-RA + DNCG + H2-RA + LT-RA**			
SM 2	→	→	→
SM 6	→	↑	↓
SM 1 (- DNCG)	→	↑	↑
**H1-RA + DNCG + H2-RA + Ibuprofen**			
SM 8	↑	↑	↓
**Prednisolone**			
SM 7	↓	↑	↑
SM 10	↑	↑	↓

MCAS: mast cell activation disease; SM: systemic mastocytosis; H1-RA: H1-receptor antagonist; H2-RA: H2-receptor antagonist; ASA: acetylsalicylic acid; DNCG: cromoglicic acid disodium salt: LT-RA: leukotriene-receptor antagonist; ↑ : increase, ↓ : decrease, or → : no significant effect after stimulation with ASA, as compared to basal release.

Substance P (SP), qualitative evaluation:

Medication & Patient	Eicosanoid		
	PGE_2	pLT	PGD_2
**H1-RA**			
MCAS 7	↓	↑	↑
SM 5	↑	↑	↑
**H1-RA + ASA**			
MCAS 8	↑	→	↓
**H1-RA + DNCG + H2-RA**			
MCAS 5	↓	↓	↓
SM 4	↑	↑	↑
SM 3	↑	↑	↑
**H1-RA + DNCG + H2-RA + LT-RA**			
SM 2	↑	→	↑
SM 6	↑	↑	↑
SM 1 (- DNCG)	↑	↓	↑
**H1-RA + DNCG + H2-RA + Ibuprofen**			
SM 8	↑	↑	↑
**Prednisolone**			
SM 7	↑	↑	↑
SM 10	↑	↑	↑

MCAS: mast cell activation disease; SM: systemic mastocytosis; H1-RA: H1-receptor antagonist; H2-RA: H2-receptor antagonist; SP: substance P; DNCG: cromoglicic acid disodium salt: LT-RA: leukotriene-receptor antagonist; ↑ : increase, ↓ : decrease, or → : no significant effect after stimulation with SP, as compared to basal release.
