# Supplementary material for: Prostaglandin D2-supplemented “functional eicosanoid testing and typing” assay with peripheral blood leukocytes as a new tool in the diagnosis of systemic mast cell activation disease: an explorative diagnostic study
Source: J Transl Med. 2014 Aug 12;12:213. doi: 10.1186/s12967-014-0213-2 (PMC4283146; doi:10.1186/s12967-014-0213-2)
Supplement: Additional file 7: — SP-triggered release of pLT from PBLs of MCAD patients. [file 12967_2014_213_MOESM7_ESM.pdf]

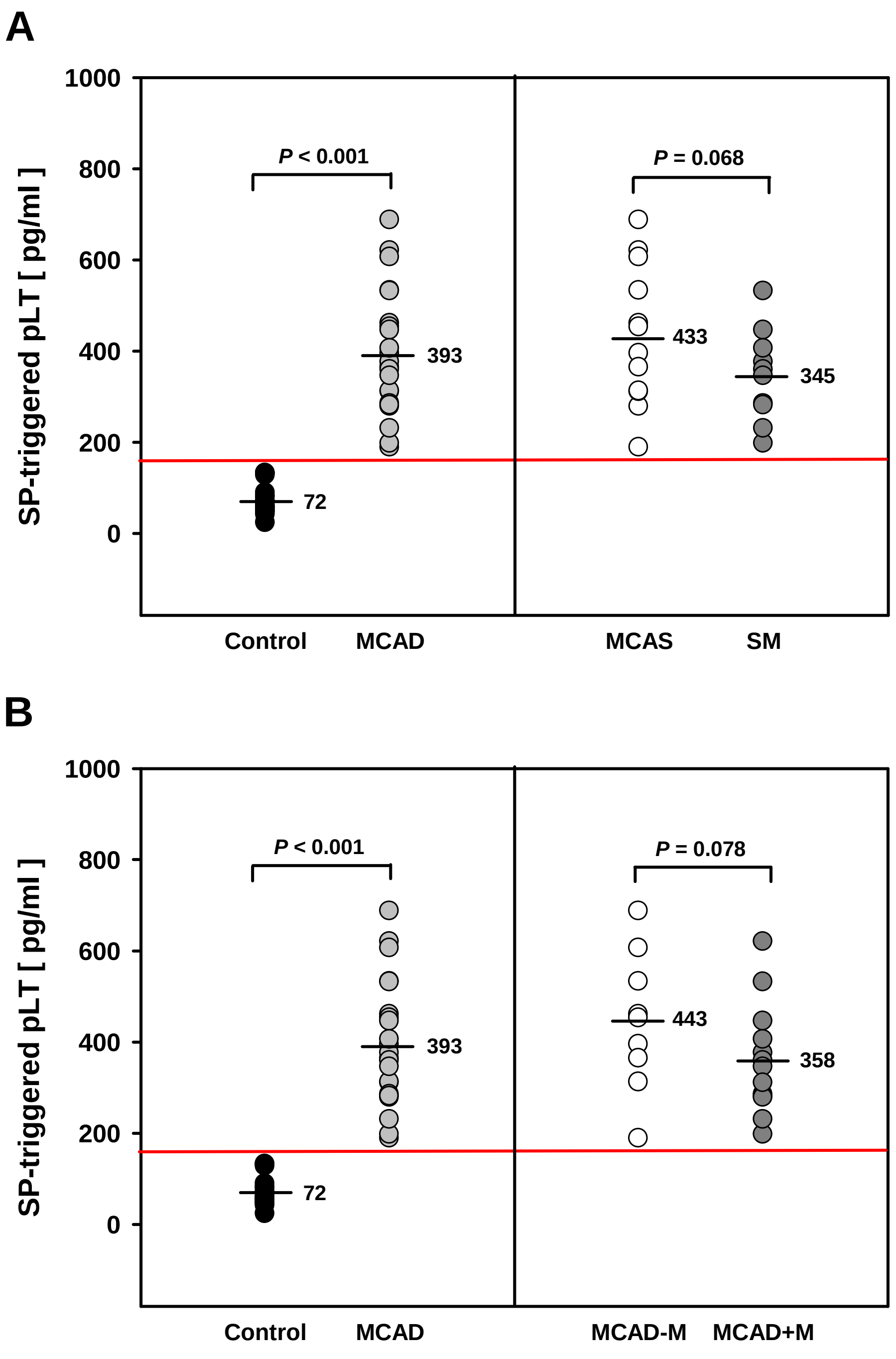

Additional file 7: **SP-triggered release of pLT from PBLs of MCAD patients.** Data represent individual pLT levels of MCAD patients and healthy controls, derived group means (black horizontal lines), and the optimal cut-off value (red horizontal line) of 159.59 pg/ml with a misclassification rate of 0.0%. Data were statistically analysed using the Student's *t*-test for unpaired values and ROC analysis, respectively. Control: healthy individuals (n = 20), MCAD: patients with mast cell activation disease (n = 22); (**A**) MCAS: MCAD patients with mast cell activation syndrome (n = 12); SM: MCAD patients with systemic mastocytosis (n = 10); (**B**) MCAD-M: MCAD patients without MCAD-specific medication (n = 9); MCAD+M: MCAD patients with MCAD-specific medication (n = 13).
